# Immune-Mediated Thrombotic Thrombocytopenic Purpura in a Patient With Systemic Lupus Erythematosus and Recent Epstein-Barr Virus Infection: A Diagnostic Challenge

**DOI:** 10.7759/cureus.110029

**Published:** 2026-06-01

**Authors:** Anna Laskova, Bohdan Syritsa

**Affiliations:** 1 Hospital Medicine, Reading Hospital, Tower Health, West Reading, USA

**Keywords:** epstein-barr virus, immune thrombocytopenia, microangiopathic autoimmune hemolytic anemia, systemic lupus erythematosus, thrombotic thrombocytopenic purpura

## Abstract

Patients with systemic lupus erythematosus (SLE) can develop thrombocytopenia from multiple etiologies. It is important not to miss immune-mediated thrombotic thrombocytopenic purpura (TTP) as a cause of severe thrombocytopenia because untreated TTP can rapidly progress to multiorgan failure and death. The diagnosis is often challenging because autoimmune cytopenias and concurrent infections may mimic TTP. We present a case of ADAMTS13-confirmed immune-mediated TTP in a patient with SLE and recent Epstein-Barr virus (EBV) infection initially suspected to have immune thrombocytopenia. Progressive microangiopathic hemolytic anemia with worsening schistocytosis and a high PLASMIC score prompted urgent initiation of plasma exchange before ADAMTS13 confirmation. The patient was successfully treated with plasma exchange, corticosteroids, rituximab, and caplacizumab.

## Introduction

Thrombotic thrombocytopenic purpura (TTP) is a rare but potentially life-threatening hematologic emergency characterized by severe deficiency of the ADAMTS13 enzyme, which cleaves large von Willebrand factor (vWF) multimers. In TTP, ultra-large vWF multimers accumulate, leading to microthrombi formation, platelet consumption, and mechanical destruction of red blood cells. This results in severe thrombocytopenia, microangiopathic hemolytic anemia (MAHA), and subsequent end-organ damage. Immune-mediated TTP is the most common form of TTP caused by acquired autoantibodies against ADAMTS13, in contrast to congenital TTP, which results from inherited mutations affecting the enzyme [1].

Systemic lupus erythematosus (SLE) is a multisystem autoimmune disease associated with a broad spectrum of hematologic abnormalities. Approximately 10-30% of patients with SLE develop thrombocytopenia due to immune-mediated and non-immune mechanisms [2]. Immune-mediated TTP is a rare but potentially fatal cause of thrombocytopenia in patients with SLE. It has been estimated to occur in approximately 1-4% of patients during the course of their disease [3]. Distinguishing immune-mediated TTP from other causes of cytopenias in SLE can be challenging, particularly in the setting of concurrent infection or overlapping autoimmune hematologic manifestations. Early recognition is critical because delayed treatment is associated with significant morbidity and mortality.

We present a case of ADAMTS13-confirmed immune-mediated TTP in a patient with SLE and recent Epstein-Barr virus (EBV) infection whose presentation initially mimicked immune thrombocytopenia (ITP).

## Case presentation

A 26-year-old woman with a history of SLE with lupus nephritis and pulmonary embolism presented to the hospital with fever, back pain, epistaxis, and gum bleeding. Her home medications included hydroxychloroquine. Physical examination was remarkable for dried blood in the left nostril and gum bleeding. Vital signs in the emergency department were as follows: temperature 37.8 °C, pulse 103 beats/min, respiratory rate 24/min, and blood pressure 148/98 mm Hg.

Pertinent laboratory test results were notable for severe thrombocytopenia, bilirubinemia, and elevated anti-double-stranded DNA antibodies. Haptoglobin was undetectable, and lactate dehydrogenase was elevated, consistent with hemolysis (Table 1).

**Table 1:** Laboratory test results on admission.

Test	Value	Reference range
White blood cells	3.8	4-10.8 x 10^9^/L
Hemoglobin	12.9	12-16 g/dL
Platelets	3	150-450 x 10^9^/L
Creatinine	0.97	0.55-1.02 mg/dL
Total bilirubin	2	0.3-1.2 mg/dL
Direct bilirubin	0.5	<0.3 mg/dL
Lactate dehydrogenase	708	120-246 IU/L
Haptoglobin	<10	30-200 mg/dL
International normalized ratio	1.1	0.9-1.1
Complement C3	62	83-193 mg/dL
Complement C4	9	15-57 mg/dL
Reticulocyte count	1.2%	0.5-2%
Anti-double-stranded DNA antibody	19	<4 IU/ml

Urine analysis revealed hematuria and proteinuria consistent with the patient's history of lupus nephritis. There were no casts.

Peripheral blood smear showed mature granulocytes without dysplasia or blasts, as well as mature lymphocytes and monocytes; schistocytes were present, and platelets were barely seen.

The direct antiglobulin test (DAT) was positive for C3 and negative for IgG. In the setting of hemolysis and severe thrombocytopenia, autoimmune cytopenias, including Evans syndrome, were considered in the differential diagnosis.

Computed tomography (CT) of the chest, abdomen, and pelvis showed diffuse axillary and abdominopelvic lymphadenopathy (Figure 1A). The study did not reveal hepatosplenomegaly (Figure 1B), although evaluation of the parenchymal organs was suboptimal because iodinated contrast was not administered due to the patient’s iodine allergy.

**Figure 1:**
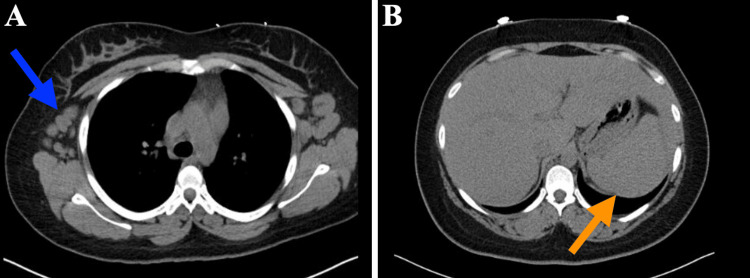
(A) CT of the chest demonstrating axillary lymphadenopathy (blue arrow). (B) CT of the abdomen demonstrating normal-appearing spleen (orange arrow).

An infectious workup was initiated because of fever, cytopenias, lymphadenopathy, and a history of immunosuppression. Pertinent positive infectious test results included a positive Monospot test, positive EBV serologies (Table 2), and a positive blood culture for Staphylococcus epidermidis in 1 of 2 bottles. Repeat blood cultures showed no growth. The tick-borne disease panel, Babesia serology, and HIV testing were negative. EBV polymerase chain reaction (PCR) was also negative. The patient received empiric ceftriaxone and vancomycin for two days; these were later discontinued because no bacterial infection was identified. The Infectious Diseases team considered the initial positive blood culture to be due to skin contamination. Based on the EBV workup, the patient was considered to be recovering from recent infectious mononucleosis.

**Table 2:** EBV antibody panel test results. EBV: Epstein-Barr virus; IgG: Immunoglobulin G; IgM: Immunoglobulin M

Test	Value	Reference range
EBV anti-nuclear antigen, IgG	>600	<18 U/ml
EBV anti-viral capsid antigen, IgM	100	<36 U/ml
EBV anti-viral capsid antigen, IgG	71.5	<18 U/ml

The Hematology and Rheumatology teams were consulted. ITP was initially suspected. The patient received intravenous immune globulin at 1 g/kg/day for two days and dexamethasone 40 mg orally daily for three days without improvement in platelet count. The patient continued to have low haptoglobin levels, elevated lactate dehydrogenase, and schistocytes, raising concern for ongoing hemolysis. Immune-mediated TTP was suspected, and ADAMTS13 activity testing was sent. The PLASMIC score was 7 (Table 3).

**Table 3:** PLASMIC score result based on the criteria described by Bendapudi et al. [4]. MCV: mean corpuscular volume; INR: international normalized ratio

Score component	Present (Yes/No)	Score value
Platelet count <30 x 10^9^/L	Yes	1
Hemolysis	Yes	1
Active cancer (treated for cancer within the past year)	No	1
History of solid-organ or stem-cell transplant	No	1
MCV <90 fL	Yes	1
INR <1.5	Yes	1
Creatinine <2.0 mg/dL	Yes	1

The patient was started on plasmapheresis and oral prednisone 80 mg daily on the fourth day of hospitalization while awaiting ADAMTS13 activity test results. Later that evening, ADAMTS13 activity was reported as undetectable, confirming the diagnosis of immune-mediated TTP. The following morning, intravenous rituximab 700 mg was administered. Caplacizumab 11 mg daily was initiated, with the first dose given intravenously and subsequent doses administered subcutaneously. Hydroxychloroquine 200 mg orally daily was continued throughout the hospitalization. The platelet count began to improve rapidly (Figure 2).

**Figure 2:**
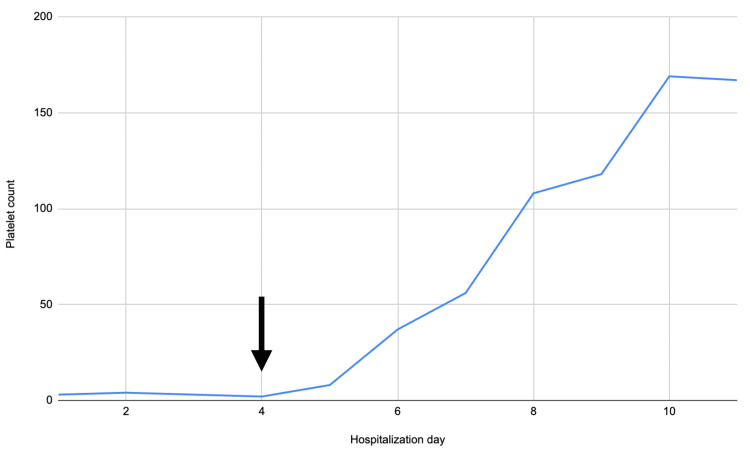
Platelet count trend during the hospitalization. Rapid platelet recovery was noted after TTP-directed management was started on the fourth day of the hospitalization (black arrow).

After normalization of the platelet count for two consecutive days, plasmapheresis was discontinued. The repeat ADAMTS13 activity was 0.71 (reference range 0.68-1.63 IU/ml). The patient reported feeling well and was discharged home on oral prednisone 80 mg daily, with plans to taper steroids in the outpatient setting. Discharge medications also included caplacizumab 11 mg subcutaneously daily, hydroxychloroquine 200 mg orally daily, and mycophenolate mofetil 500 mg orally twice daily. The patient was scheduled for outpatient follow-up with the Hematology and Rheumatology teams.

## Discussion

Our patient with established SLE presented with severe thrombocytopenia, mucosal bleeding, fever, diffuse lymphadenopathy, and evidence of recent EBV infection. Additional laboratory findings included mild anemia, hemolysis, and DAT positivity. Because ITP is among the most common causes of thrombocytopenia in SLE [2], the standard therapy with intravenous immune globulin and corticosteroids was initiated. Furthermore, the patient lacked several classic features of TTP, including significant end-organ injury or profound anemia. Fewer than 10% of patients with TTP present with the full clinical pentad of thrombocytopenia, MAHA, neurologic abnormalities, fever, and renal failure [1], and our patient had no neurologic or renal involvement at presentation.

Elevated anti-double-stranded DNA antibody levels and decreased complement levels supported active SLE. Hematuria and proteinuria were consistent with known lupus nephritis. However, the normal creatinine level and the severity of thrombocytopenia were not characteristic of a lupus nephritis flare [5].

DAT positivity further complicated the diagnostic evaluation by raising concern for autoimmune hemolytic anemia (AIHA) or Evans syndrome. Evans syndrome is a rare, severe disease characterized by the combination of AIHA and ITP [6]. However, progressive schistocytosis, absence of reticulocytosis, and severe thrombocytopenia were more consistent with a microangiopathic process rather than primary AIHA. Importantly, DAT positivity does not exclude immune-mediated TTP, particularly in patients with underlying SLE. Complement-mediated DAT positivity can be observed in SLE due to immune dysregulation and complement activation, most commonly with isolated C3 deposition [7]. In the absence of clinical features typical of warm AIHA (prominent spherocytosis and marked reticulocytosis), isolated C3 positivity was interpreted in the context of SLE rather than primary antibody-mediated hemolysis in this patient.

The patient failed to respond to corticosteroids and intravenous immunoglobulin and continued to demonstrate progressive schistocytosis, persistent severe thrombocytopenia, and ongoing hemolysis, raising concern for evolving MAHA rather than isolated ITP or Evans syndrome. These findings, together with a PLASMIC score of 7, which predicts severe ADAMTS13 deficiency with high specificity [4], prompted urgent initiation of plasma exchange while awaiting ADAMTS13 activity testing, which later confirmed immune-mediated TTP.

Evidence of recent EBV infection supported an infectious or post-infectious etiology for the patient’s presentation, as EBV can be associated with fever, lymphadenopathy, and thrombocytopenia. However, EBV-related thrombocytopenia is typically mild and self-limited [8]. The degree of refractory cytopenia in this case was not fully explained by EBV infection alone.

Recent EBV infection may have contributed to the development of immune-mediated TTP in this patient with underlying SLE. Viral infection is a recognized precipitating factor for immune-mediated TTP. Among viral infections, HIV has been most commonly associated with TTP [9], whereas EBV-associated immune-mediated TTP has been reported rarely [10]. EBV infection can induce polyclonal B-cell activation and autoantibody production, providing a plausible immunologic mechanism for the development of anti-ADAMTS13 autoantibodies in susceptible individuals [11].

After ADAMTS13 activity returned undetectable, rituximab and caplacizumab were added to the management plan. The patient had an excellent clinical response.

## Conclusions

In patients with SLE presenting with severe thrombocytopenia, hemolysis, and schistocytosis, immune-mediated TTP should remain a key diagnostic consideration even in the presence of DAT positivity or concurrent infection. Early recognition and prompt initiation of plasma exchange-based therapy are critical to improving outcomes.
